# Frequency of Diplopia in Zygomatic Complex Fractures—A Cross-Sectional Descriptive Study

**DOI:** 10.1155/2023/7631634

**Published:** 2023-11-08

**Authors:** Maria Shabbir, Ruqaya Shah, Muhtada Ahmad, Rakhi Issrani, Zafar Khan, Salah Nazal Alotha, Basant Mousa Alsiyat, Mohammed Saad Alqarni, Ahmed Saleh Albalawi, Namdeo Prabhu, Mohammad Khursheed Alam, Zahid Qayyum

**Affiliations:** ^1^Department of Oral & Maxillofacial Surgery, Shaheed Muhtarma Benazir Bhutto Institute of Trauma, Karachi, Pakistan; ^2^Department of Oral & Maxillofacial Surgery, Jinnah Postgraduate Medical Centre, Karachi, Pakistan; ^3^Department of Oral & Maxillofacial Surgery, Dow Dental College, Dow University of Health Sciences, Karachi, Pakistan; ^4^Department of Preventive Dentistry, College of Dentistry, Jouf University, Sakaka, Saudi Arabia; ^5^Frontier Medical and Dental College, Abbottabad, Pakistan; ^6^College of Dentistry, Jouf University, Sakaka, Saudi Arabia; ^7^Department of Oral & Maxillofacial Surgery and Diagnostic Sciences, College of Dentistry, Jouf University, Sakaka, Saudi Arabia; ^8^Department of Dental Research Cell, Saveetha Institute of Medical and Technical Sciences, Saveetha Dental College and Hospitals, Chennai, India; ^9^Department of Public Health, Faculty of Allied Health Sciences, Daffodil International University, Dhaka, Bangladesh; ^10^Department of Oral & Maxillofacial Surgery, Khyber Girls Medical College, Hayatabad Medical Complex, Peshawar, Pakistan

## Abstract

**Background:**

The zygomatic complex is the second most common fracture of the facial bones after the nasal bone. The prominent convex shape of the zygoma makes it vulnerable to traumatic injury. Diplopia is one of the serious complications of zygomatic complex fracture and is a common subjective complaint.

**Objective:**

To determine the frequency of diplopia in zygomatic complex fractures. *Methodology*. A cross-sectional descriptive study was conducted at the Oral and Maxillofacial Surgery Ward, Civil Hospital, Karachi, Pakistan. The duration of the study was 1 year (March 1, 2021 to February 28, 2022). A total of 126 patients having zygomatic complex fractures were included in this study. After recording the patient's complete history, like demographic details and cause for fracture, diplopia was examined clinically. If, during the examination, the patient complained of double vision, this was labeled as diplopia positive (Yes) and negative (No) if the patient did not have any such complain. Data were statistically analyzed.

**Results:**

The mean (±SD) age of patients was 33.42 (±9.27), with 91 (72.2%) male patients and 35 (27.8%) female patients. The frequency of diplopia in zygomatic complex fractures was observed in 52 (41.3%) patients. The rate of diplopia was significantly high in patients aged between 31 and 40 years (*P*-value=0.0005).

**Conclusion:**

The frequency of diplopia among patients having zygomatic complex fractures was high in this study. Thus, forming a strategy to properly diagnose and treat it and to prevent persistent morbidity to improve patient's quality of life is recommended.

## 1. Introduction

Maxillofacial injuries are common in Pakistan and are associated with a high incidence of facial fractures in different combinations [1]. Fractures involving the zygomatic complex are the second most common fractures of the facial bones after the nasal bone. The prominent convex shape of the zygoma makes it vulnerable to traumatic injury [2]. The etiology of the zygomatic complex fractures includes road traffic accidents, assaults, falls, and sports injuries [3]. Minimally displaced zygomatic complex fractures can result in functional and esthetic deformities [2]. Even minor injuries can cause considerable morbidity and time lost from work [4].

Zygomatic complex fractures are frequently complicated by injury to the orbit and eye adnexae, which are the most serious negative outcomes of this kind of fracture [2, 5–8]. All traumas to the face above the level of the oral cavity require careful ocular examination, including an estimation of the visual acuity of each eye. Diplopia is one of the serious complications of zygomatic complex fracture and is a common subjective complaint [2]. Traumatic diplopia is broadly variable in time and extent. Two varieties of diplopia exist, and it is important to distinguish between them. Monocular diplopia or blurring of the vision through one eye with the other closed, requires the immediate attention of an ophthalmologist sine it usually indicates a detached lens, hyphema, or other traumatic injury to the globe. Binocular diplopia is the other one in which the blurring of vision occurs only when the patient looks through both eyes simultaneously. It is common and occurs in approximately 10.0% to 40.0% of zygomatic complex injuries [9, 10]. Furthermore, diplopia may be transient or persistent. Inadequate diagnosis and treatment at improper times and tethering or fibrosis of muscles may lead to persistent diplopia [8, 11–15].

Although diplopia is a serious eye injury but studies on diplopia, especially after zygomatic complex fractures for which specialist assessment and management are required, are lacking in our region, and also there is a paucity of evidence in this part of our country. Furthermore, we know that ophthalmic assessments of patients presenting at emergency clinics are often missed by nonophthalmic personnel/specialists, and due to this, there is variability in the prevalence of diplopia in various studies. Hence, the epidemiological data obtained from this study will help to create awareness among the maxillofacial surgeons to assess diplopia among patients having zygomatic complex fracture at presentation by simple clinical examination and facilitate the patients to undergo specialist opinions, investigations, and treatment strategies as early as possible to prevent persistent morbidity and improve the quality of life of the patients.

The aim of this study is to determine the frequency of diplopia in zygomatic complex fractures.

## 2. Materials and Methods

### 2.1. Study and Sample Characteristics

A cross-sectional descriptive study was conducted at wards of the Oral and Maxillofacial Surgery, Civil Hospital, Karachi, Pakistan. Approval of data collection was sought from the institutional ethical review board of Dow University of Health Sciences, Karachi. The duration of the study was 1 year (March 1, 2021 to February 28, 2022). A sample size of 126 cases was calculated with 95% confidence level, 13% margin of error, and taking expected percentage of diplopia in 9.0% cases in patients with zygomatic complex fracture [2]. The sample was selected using nonprobability, consecutive sampling technique.

### 2.2. Inclusion Criteria

Patients of genders aged 18–50 years and those having zygomatic complex fractures and presented within the first 24 hr of injury were included in the study.

### 2.3. Exclusion Criteria

Patients with significant psychiatric disorder, intoxicated, unconscious, sedated, or intubated that were not able to provide reliable information, having isolated zygomatic arch fractures or isolated nasoethmoid fractures or panfacial fractures and with previous history of diplopia were excluded from the study.

### 2.4. Procedure

In total, 126 patients presenting with zygomatic complex fractures were assessed and examined for the presence of diplopia within the first 24 hr of injury as mentioned in the inclusion criteria were included in the study through wards of the Department of Oral and Maxillofacial Surgery, Civil Hospital, Karachi. Informed consent was obtained before the procedure. Complete history from the patients having zygomatic complex fractures was enquired, including patient's demographics like age and gender and etiology of zygomatic complex fractures. Diplopia was examined clinically, and if at any point of examination, the patient complaint of double vision, then this was labeled as diplopia positive (Yes) or negative (No) if the patient did not have any such complaint. Patients were evaluated by a well-qualified and an experienced senior consultant surgeon.

### 2.5. Statistical Analysis

Statistical analysis was performed with SPSS software version 20.0 (IBM Corp, Armonk, NY, USA). Mean ± SD was computed to present quantitative variable like age. Frequency and percentages were calculated for all qualitative variables, such as gender, etiology of fracture, and diplopia. Effect modifier was controlled through stratification of age, gender, and etiology of fracture to see the effects of these on outcome variables applying *X*^2^ test taken *P* ≤ 0.05 as significant.

## 3. Results

Table 1 shows the summary of descriptive statistics of the studied participants. A total of 126 patients having zygomatic complex fractures were included in this study. The majority of the patients belonged to the age group of 31–40 years (57; 45.2%). Out of 126 cases, 91 (72.2%) were male and 35 (27.8%) were female. Regarding the etiology of fracture, 65 (51.6%) were injured in road traffic accidents, 43 (34.1%) were injured due to falls, and 18 (14.3%) were assaulted. The right eye was affected in 55 (43.7%) cases, and the left eye was affected in 71 (56.3%) cases. The frequency of diplopia in zygomatic-complex fractures was observed in 52 (41.3%) cases.

Diplopia was significantly high in patients between 31 and 40 years of age (*P*-value = 0.0005). The frequency of diplopia was insignificant among the genders and for different etiologies (*P*-value = 0.164 and 0.43, respectively).  Table 2 shows the frequency of diplopia in zygomatic complex fracture with respect to age groups, gender, and etiology of fracture.

**Figure 1:**
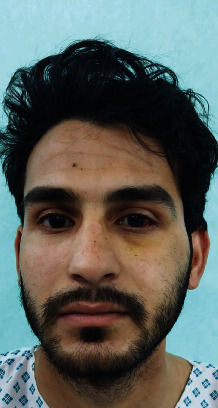
Posttraumatic and preoperative frontal profile picture of the 24-year-old male patient presenting with left zygomatic complex fracture, conjunctival hemorrhage, enophthalmos, and diplopia.

**Figure 2:**
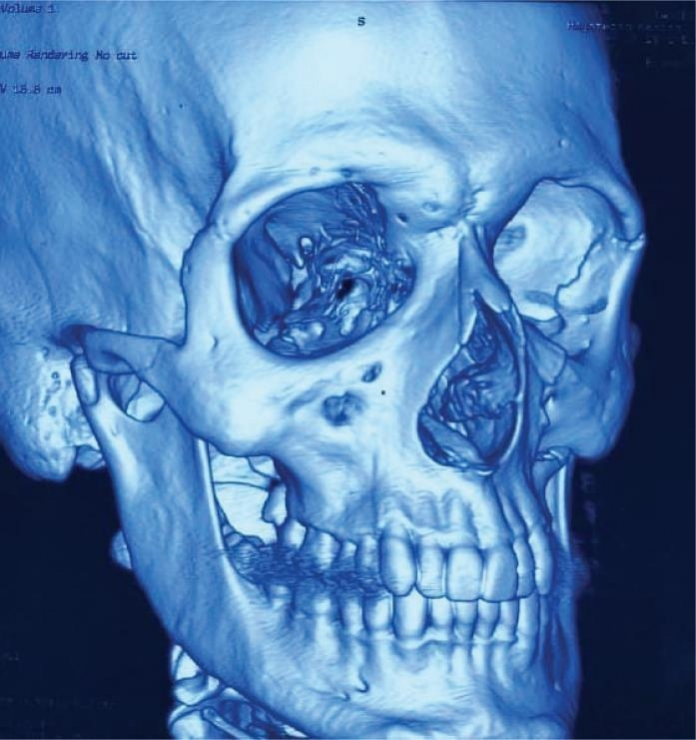
3D CT scan delineating the infra-orbital rim fracture and involvement of the orbital floor.

**Figure 3:**
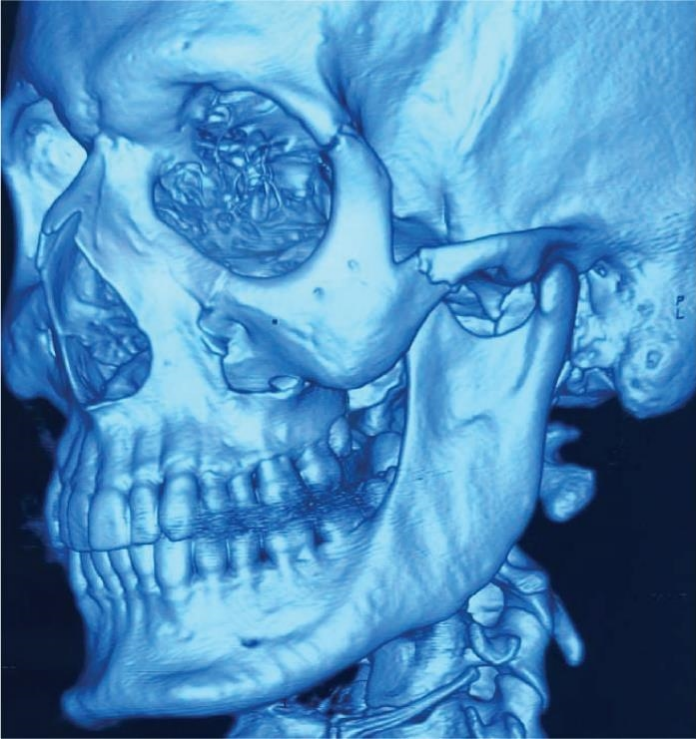
3D CT scan showing the left zygomatic complex bone fracture involving the fronto-zygomatic, zygomatico-maxillary, and zygomatico-temporal suture along with the orbital floor.

**Figure 4:**
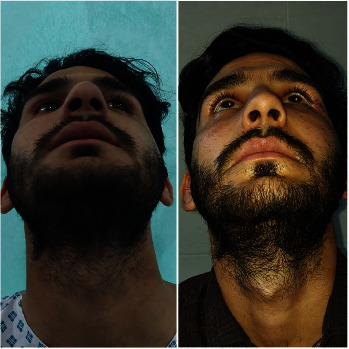
Pre- and postoperative pictures of the patient with a zygomatic complex fracture and after open reduction and internal fixation and correction of enophthalmos and diplopia.

**Figure 5:**
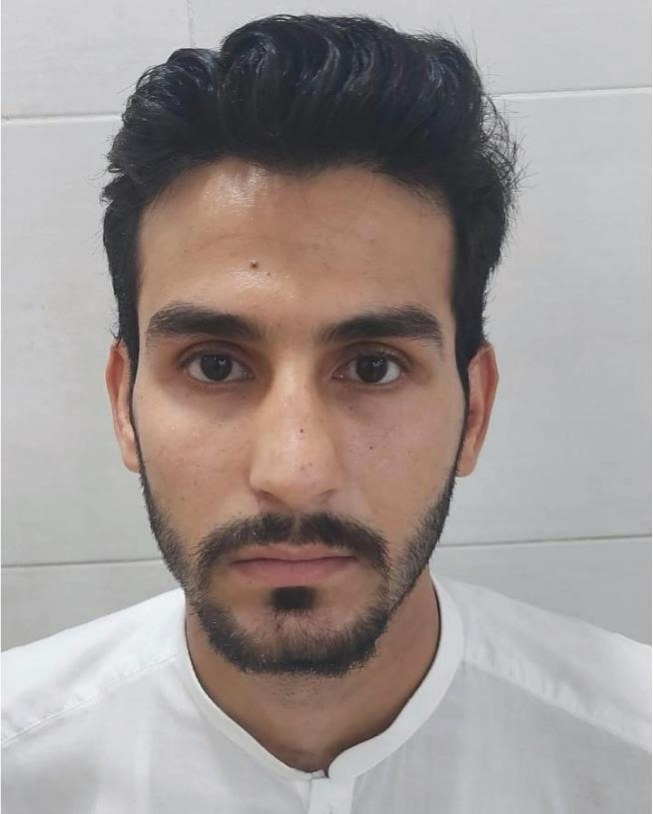
One-month postoperative frontal view of the same patient.

Figures 1–5 show the preoperative and postoperative clinical and radiographic images of a patient who presented with a left zygomatic complex fracture, conjunctival hemorrhage, enophthalmos, and diplopia.

## 4. Discussion

The zygomatic complex gives the cheek prominence, and it is the second most common mid-facial bone fracture after the nasal bone and overall represents 13.0% of craniofacial fractures [16]. Zygomatic complex fractures are almost always associated with fractures of the floor of the orbit. Typically, a fracture line extends from the inferior orbital fissure anteromedially along the orbital process of the maxilla toward the infraorbital rim. Facial bones, especially of the middle third of the face, are composed of a network of fragile bones held together across sutures, which give way in case of force to a lesser extent than other parts of the body. The key management of facial trauma is to operate the cases as soon as clinical conditions permit, with a special emphasis on function and esthetics [17].

In this study, the average age of the patients was 33.42 ± 9.27 years. Out of 126 cases, 91 (72.2%) were males and 35 (27.8%) were female. Regarding the etiology of fracture, 65 (51.6%) were injured in road traffic accidents, 43 (34.1%) were injured due to falls, and 18 (14.3%) were assaulted. The result of our study is comparable with those reported by Van Hoof et al. [18], Zachariades and Papavassiliou [19], Tanaka et al. [20], Bataineh et al. [21], and Kreutziger and Kreutziger [22], which shows the road traffic accidents were the most common cause of facial fractures.

The high number of maxillofacial injuries due to road traffic accidents in our country is due to the lack of road sense among the road users, overspeeding, under-age drivers, slow-moving vehicles on roads like tractor trolleys, etc., bad condition of vehicles, overloading and bad condition of roads. A large number of people belong to the low socioeconomic group in this region of the country, and they use public transport vehicles operated by illiterate road sense drivers, which leads to accidents; that is the one reason for the high number of road traffic accidents cases. The developed countries strictly follow the road traffic regulations, well trained public transport drivers, and well arrange seating capacities of public transport plus the strict seat belt legislation is also strictly followed, which leads to a reduction in road traffic accidents.

In the present study, the frequency of diplopia in zygomatic complex fractures was observed in 52 (41.3%) cases. A similar result was reported by Amrith et al. [23], where they found significant diplopia in 40.0% of patients. On the contrary, Obuekwe et al. [24] reviewed 134 cases of this type of fracture and found that only 9.0% of cases have developed diplopia. Khreisat [2], in his study, reported an incidence of 18.0% of diplopia. Qayum et al. [25] found diplopia to be present in 27.5% of 40 patients. It is worth noting that most of the patients in this study had diplopia immediately after the injury, whereas few developed diplopia within 4–6 hr after trauma.

In the study conducted by de Man et al. [26], out of nine patients with diplopia, five patients had exploration and treatment for the orbital floor within 8–13 days of presentation, and three patients were treated after 14 days while one patient received treatment within 4–7 days following injury. They recommended that if vertical limitation exists with large herniation of orbital contents into the maxillary sinus, a “wait and see” policy should be employed to allow resolution of the initial hemorrhage and residual edema. If, following examination, eye movements improve, then the operation should be delayed. This policy was adopted by the maxillofacial surgeons at the Manchester Royal Infirmary. According to the findings of Egbert et al. [27], there was a negative association between the return of ocular motility and resolving of diplopia if there is a delay in surgery. Early surgery resulted in not only more rapid improvement of preoperative motility deficit and diplopia but also improvement in visual acuity. The patients of this study received treatment wherever indicated.

Diplopia is a shared clinical feature in mid-facial trauma, particularly in orbital and zygomatic complex fractures. Zygomatic complex fractures are regularly convoyed by orbital fractures, with various degrees of comminution of the orbital floor and herniation into the maxillary sinus. They are possibly the most common fractures that involve the orbit. Conspicuously, elusive orbital floor fractures are often times missed by maxillofacial surgeons but have substantial consequences. Operating surgeons must, therefore, undertake vigilant ophthalmic assessment in zygomatic complex fractures to avoid complications [28].

## 5. Conclusion

The frequency of diplopia among patients having zygomatic complex fractures was high in this study. Thus, forming a strategy to properly diagnose and treat it and to prevent persistent morbidity to improve patient's quality of life.

## Figures and Tables

**Table 1:** Summary of descriptive statistics (*N* = 126).

	*N* (%)
Age groups (years)	
≤20	17 (13.5)
21–30	27 (21.4)
31–40	57 (45.2)
≥41	25 (19.9)
Mean age (years)	
Gender distribution	33.42 ± 9.27
Male	91 (72.2)
Female	35 (27.8)
Etiology of fracture	
Road traffic accidents	65 (51.6)
Falls	43 (34.1)
Assaults	18 (14.3)
Side of eye affected	
Right eye	55 (43.7)
Left eye	71 (56.3)
Frequency of diplopia in zygomatic-complex fractures	
Present (yes)	52 (41.3)
Absent (no)	74 (58.7)

**Table 2:** Frequency of diplopia in zygomatic complex fracture with respect to age groups, gender, and etiology of fracture.

Variable		Diplopia		*P*-value (*X*^2^ test)
		Yes (*N* = 52)	No (*N* = 74)	
Age groups (years)	≤20	02 (11.8%)	15 (88.2%)	0.0005 ^*∗*^
	21–30	04 (14.8%)	23 (85.2%)	
	31–40	28 (49.1%)	29 (50.9%)	
	≥41	18 (72.0%)	07 (28.0%)	

Gender	Male	41 (45.1%)	50 (54.9%)	0.164
	Female	11 (31.4%)	24 (68.6%)	

Etiology of fracture	Road traffic accidents	29 (44.6%)	36 (55.4%)	0.43
	Falls	18 (41.9%)	25 (58.1%)	
	Assaults	05 (27.8%)	13 (72.2%)	

*^*∗*^Statistically significant*.

## Data Availability

The dataset used in the current study will be made available on request from Dr. Maria Shabbir; drmariashabbir@hotmail.com.

## Additional Points

*Clinical Significance*. This study can help to create awareness among the maxillofacial surgeons to assess diplopia among patients having zygomatic complex fracture at presentation by simple clinical examination and facilitate the patients to undergo specialist opinions and treatment.
